# Arbuscular mycorrhizal fungi in soil and roots respond differently to phosphorus inputs in an intensively managed calcareous agricultural soil

**DOI:** 10.1038/srep24902

**Published:** 2016-04-22

**Authors:** Wei Liu, Yunlong Zhang, Shanshan Jiang, Yan Deng, Peter Christie, Philip J. Murray, Xiaolin Li, Junling Zhang

**Affiliations:** 1 Centre for Resources, Environment and Food Security, College of Resources and Environmental Sciences, China Agricultural University; Key Laboratory of Plant-Soil Interactions, Ministry of Education, Beijing 100193, China; 2 College of Landscape and Art, Jiangxi Agricultural University, Nanchang 330045, China; 3 Sustainable Soil and Grassland Systems, Rothamsted Research, North Wyke, Okehampton, EX20 2SB, UK

## Abstract

Understanding the diversity and community structure of arbuscular mycorrhizal fungi (AMF) is important for potentially optimizing their role in mining phosphorus (P) in agricultural ecosystems. Here, we conduct a comprehensive study to investigate the vertical distribution of AMF in a calcareous field and their temporal structure in maize-roots with fertilizer P application over a three-year period. The results showed that soil available-P response to P fertilization but maize yields did not. Phosphorus fertilization had no-significant effect on richness of AMF except at greater soil-depths. High P-supply reduced root colonization while optimum-P tended to increase colonization and fungal richness on all sampling occasions. Crop phenology might override P-supply in determining the community composition of active root inhabiting fungi. Significant differences in the community structure of soil AMF were observed between the controls and P treatments in surface soil and the community shift was attributable mainly to available-P, N/P and pH. Vertical distribution was related mainly to soil electrical conductivity and Na content. Our results indicate that the structure of AMF community assemblages is correlated with P fertilization, soil depth and crop phenology. Importantly, phosphorus management must be integrated with other agricultural-practices to ensure the sustainability of agricultural production in salinized soils.

Low phosphate (P) availability is a major factor constraining plant growth and metabolism in many soils worldwide due to the poor solubility and mobility of soil P. As a consequence, increasing the application rates of P in the form of inorganic fertilizers or P-rich organic manures is a very important approach to overcoming soil P deficiency and achieving higher crop yields. For example, the consumption of chemical P fertilizers in China has increased approximately 100 times between 1960 (0.05 Mt) and 2010 (5.3 Mt) but yields have not increased at the same rate. Most fertilizer P applied to the soil is immobilized due to the strong adsorption of P to iron and aluminum cations at low pH and calcium (Ca) at high soil pH^1^. The recovery of applied P by crops in one growing season is often low. Average P use efficiency is estimated at <20% of fertilizer P applied in China^2^. However, excessive P inputs and inappropriate P management are found in both crop and animal production systems throughout the country, resulting in severe eutrophication of surface waters^3^. From 1980 to 2007 an average of 242 kg P ha^−1^ has accumulated in the soils, resulting in an increase in average Olsen-P levels of 7.4 to 24.7 mg kg^−1 ^4. This situation prevails even though rock phosphate is a non-renewable and finite resource and costs of fertilizer P are increasing. It is estimated that the P reserves in China (3700 million tonnes) will be exhausted within the next 20 years unless either current rates of extraction are reduced^5^ or the efficiency of the extraction technology is increased. Hence, appropriate P management to increase P use efficiency and minimize damage to the environment is of strategic importance for the development of sustainable agriculture.

Two effective strategies for P management have been developed, namely soil-based P management to optimize the use of P based on yield response to fertilizer P^6^ and plant/rhizosphere-based P management which aims to modify rhizosphere processes by localized nutrient supply, intercropping and exploitation of plant genetic potential through conventional and molecular breeding^7^. Soil microorganisms are vital constituents of the rhizosphere and they play key roles in P cycling^8^. Arbuscular mycorrhizal fungi (AMF) are members of the Glomeromycota, a key component of the soil microbiota which form the most common and widespread terrestrial plant symbioses. They are obligate symbiotic soil fungi and they form intimate associations with approximately 80% of terrestrial plant species including the majority of agricultural crops^9^. AM fungi have been shown to benefit crop productivity due to their contribution to plant nutrition, soil structure and other ecosystem services^9^. The predominant function of AMF is attributed to increased host plant P uptake as a consequence of their high affinity P uptake mechanism^10^. There is, however, great concern that high soil P contents reduce the benefit, especially in high-input agricultural systems. The potential to incorporate the management of AMF as a ‘green tool’ to maximize crop P use efficiency, reduce fertilizer P use and increase crop yields has been highlighted by the looming crisis in P reserves and increasing awareness of sustainable nutrient management^11^. It has been estimated that inoculation with AMF might result in a reduction of approximately 80% of the recommended fertilizer P rates under certain conditions^12^.

An improved understanding of AMF community responses to P fertilization is fundamental for better P management and more effective fertilizer use in agricultural ecosystems. It is generally acknowledged that the formation and growth of AM is adversely affected by high P supply levels when P is applied homogenously to the soil in pot experiments under controlled conditions^13^. However, under field conditions the influence of fertilizer P on AMF community structure and abundance is variable. For example, the abundance or diversity of AM fungi was reduced by P fertilization^14^, but application of different forms of organic and inorganic P fertilizers did not affect soil or root-inhabiting AMF over a three-year period in a maize-soybean rotation^15^. Recent evidence indicates that high soil P supply does not always have a negative impact on AMF diversity^16^. Discrepancies between different experiments may be attributed to various factors including P application rate, soil properties and climatic conditions. In addition, spatial heterogeneity is an important determinant of the AMF community. Patchy distribution of soil properties in agricultural fields has strong effects on the community structure of AMF^17^. A previous study shows that deeper soils are rich in AMF diversity^18^. As fertilizer P is typically applied to the topsoil and may thus create P hotspots, the question remains as to whether or not the AMF community in deeper soils is less responsive to fertilizer P inputs.

AMF are widely distributed and knowledge of how spatial factors affect their diversity in a specific area or a specific soil type is essential for good management^15^ because changes in available P vary greatly in different management zones^19^. For example, the accumulation of Olsen-P led to a surplus of 100 kg P ha^−1^ yr^−1^. This equates to 6 mg P kg^−1^ soil in northeast China but only 3 mg P kg^−1^ soil in north China^20^. The North China Plain (NCP), one of the most important agricultural production regions in China, provides more than 75% of the national wheat crop and 35% of the maize^21^. The net P surplus in this region is 53 kg ha^−1^ yr^−1^ (total P input 92 kg ha^−1^ yr^−1^ and total P agronomic output 39 kg ha^−1^ yr^−1^) with yield increases of wheat and maize from 0.6 and 0.7 t ha^−1^ to 5.4 and 5.6 t ha^−1^, respectively, from 1949 to 2009^22^. Hence, systematic investigations into AMF communities in soils and crop roots in response to P fertilization might allow us to better understand the potential functioning and benefits of AMF. Results from long-term experimental sites show that P fertilization decreased AMF diversity and shifted community structure^23^. However, short-term studies on AMF responses to P fertilization gradients are also necessary for improved management practices including P fertilization regimes in order to avoid the occurrence of long-term negative effects. In the present study we investigated the vertical distribution of the soil AMF community at maize harvest at a field experimental site on the North China Plain. Phosphorus fertilizer had been applied to the soil for three years. Plant AMF community composition differs from that in the soil^24^ and therefore shifts in the AMF communities in maize roots were also assessed throughout the growing season. It is difficult to find soils that are low in background P in this intensive agricultural region and the study was therefore conducted in a soil of moderate P status. We tested the hypothesis that high P supply reduces AMF diversity and that the negative impacts due to P fertilization may be more pronounced in the topsoil than deeper in the soil profile. We also predicted that maize growth stage may interact with P fertilization to influence the root AM community.

## Results

### Maize yields and soil physico-chemical properties

Due to the high application rates of fertilizer P by local farmers prior to the start of the experiment, maize yields were not significantly increased by fertilizer P inputs over the three years of the experiment. The mean average yield in the sampling year in the control (6.74 t ha^−1^) tended to be lower than in P25 (6.91 t ha^−1^) or P100 (7.15 t ha^−1^). However, shoot P concentrations were significantly affected with 2.56 (P0), 3.04 (P25) and 3.21 g kg^−1^ (P100) at the V6 growth stage. The corresponding values at the V13 stage were 1.76, 2.32 and 2.56 g kg^−1^. In contrast to the poor plant growth responses, P fertilization had significant effects on soil Olsen-P (*p* < 0.001), pH (*p* < 0.01) and EC (*p* < 0.001) (Table S1). The Olsen-P concentration in the top 20 cm of the soil profile increased significantly with fertilizer P application. The available P contents at P25 and P100 were 2.31 and 5.24 times higher than that of the control (Table S1). A significant decrease in soil pH and increase in EC were detected at P100 at all soil depths and the effects were more pronounced at 0–20 cm depth. However, no significant difference was observed between the control and P25 at any soil depth. Soil depth had significant effects on soil physico-chemical properties (Table S1). The EC and Na concentration increased significantly with increasing soil depth (*p* < 0.001) and the other parameters (total N, SOM, C/N, Olsen-P, Zn) deeper in the soil profile (20–40 and 40–60 cm) were generally lower (*p* < 0.001) than those at the top 20 cm. Soil N/P appeared to decrease with P application and increased with soil depth, and the effect of soil depth was more pronounced in P fertilized treatments than in the control. Soil available K and microelements (Fe, Mn and Cu) were not significantly affected by either fertilizer P or soil depth (data not shown).

### AM colonization and hyphal growth

Root length colonization (%RLC) and arbuscular colonization (%AC) in maize roots were strongly influenced by both growth stage and P fertilization (Table 1). In general, the high P rate (P100) decreased %RLC, %AC, and %HC (hyphal colonization) while the effect of optimum P (P25) was variable and an increase in colonization was observed at certain growth stages. The %RLC increased over the growing season and the highest value was observed at R4. The %AC ranged from 14 to 28% and had the highest values at V13. The percentage of vesicles was low and affected by neither growth stage nor fertilizer P. The %HC was variable and was strongly influenced by growth stage but not by fertilizer P. Fertilizer P reduced %HC at V6 and V13 but the low rate of P (P25) increased %HC at the R4 stage. Hyphal length density was affected by neither growth stage nor fertilizer P. Spore density tended to decrease at R4 and the effect was stronger at P100 than in the control or P25.

### Structure of the soil AM fungal community

**Figure 1:**
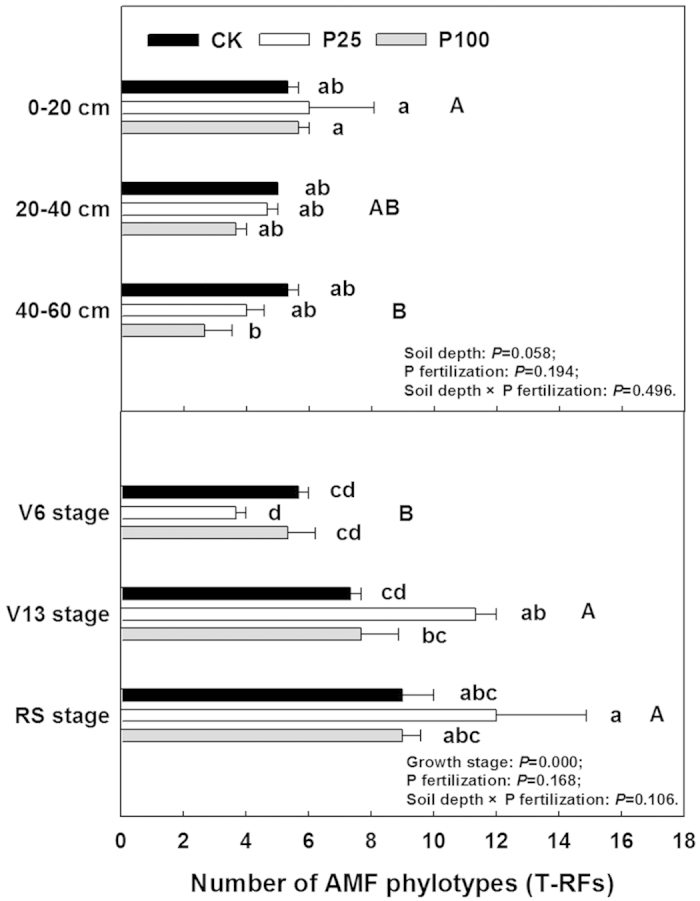
Richness of fungal T-RFs in soil at different depths and maize roots of different growth stages in different P treatments. Bars represent mean values ± SE (n = 4). Significant differences among treatments and soil depths were tested using Duncan’s multiple range test (*p* < 0.05) and are indicated by different lowercase or capital letters.

The P application rate did not have any significant effect on soil AMF richness except at 40–60 cm soil depth where richness decreased significantly at P100 (Fig. 1). The T-RF richness in the two P treatments tended to decrease deeper in the soil profile compared to 0–20 cm depth. T-RFLP analyses in combination with cloning and sequencing were used to determine the AM fungal community. Thirteen T-RFs (97, 107, 116, 140, 141, 142, 157, 168, 169, 189, 190, 258 and 259 bp) were detected in T-RFLP profiles (Table S2). Three T-RFs (107, 142 and 157 bp) showed lower frequency of occurrence, while five T-RFs (116, 169, 141, 190 and 259 bp) ranked in the top five and accounted for approximately 76.2% of total T-RF abundance (Table S2 and Fig. S1). The T-RFLP fingerprints show that the fungal community was greatly changed by P fertilization and varied with soil depth (Table S2). The fungal community at P100 differed greatly from that at P25 while the latter showed a fungal community shift only at the top 20 cm of the soil profile compared to that in the control.

The T-RFs of 169 bp and 116 bp (except 40–60 cm depth in P100) were present in all soil samples and the other common T-RFs (141, 190 and 259 bp) were more frequently detected in the control and P25. 141 bp was only detected at all soil depths in the control and P25, and other T-RFs (97, 107, 140, 142, 157, 168 and 258 bp) were detected only in P fertilizer treatments at certain soil depths.

**Figure 2:**
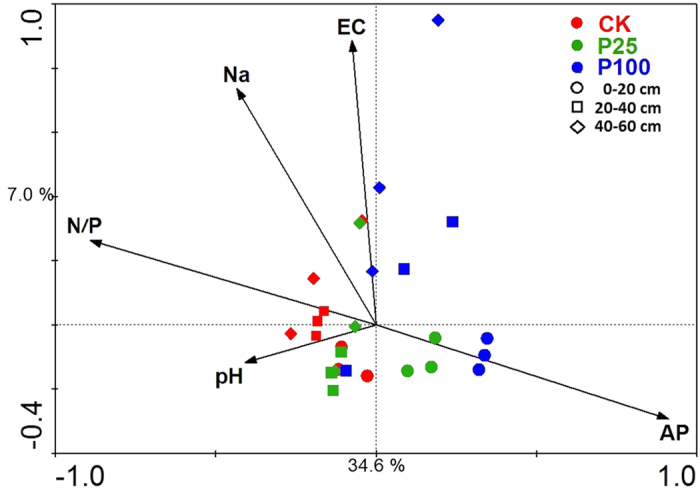
Canonical correspondence analysis (CCA) of the AM fungal community composition in the soil in response to vectors of significant soil chemical properties. CK: zero P application (control); P25: 25 kg P ha^−1^; P100: 100 kg P ha^−1^. Solid circles represent 0–20 cm, solid squares represent 20–40 cm, and solid diamonds represent 40–60 cm soil depth, respectively. The first and second axes explain 34.2 and 7.0% of the variance. The Monte Carlo test of significance of the first canonical axis and all canonical axes are *p* = 0.002 (*F* = 6.238) and *p* = 0.002 (*F* = 2.372), respectively.

The significance of soil chemical variables in relation to the soil T-RFLP profiles was explored using CCA (Fig. 2). Soil AP, N/P and pH showed significant effects on the AMF community, while Na and EC were positively correlated with T-RFs down the soil profile. There was a clear separation of AMF community between 40–60 cm depth and other two soil depths.

### Structure of AM fungal community in maize roots

**Figure 3:**
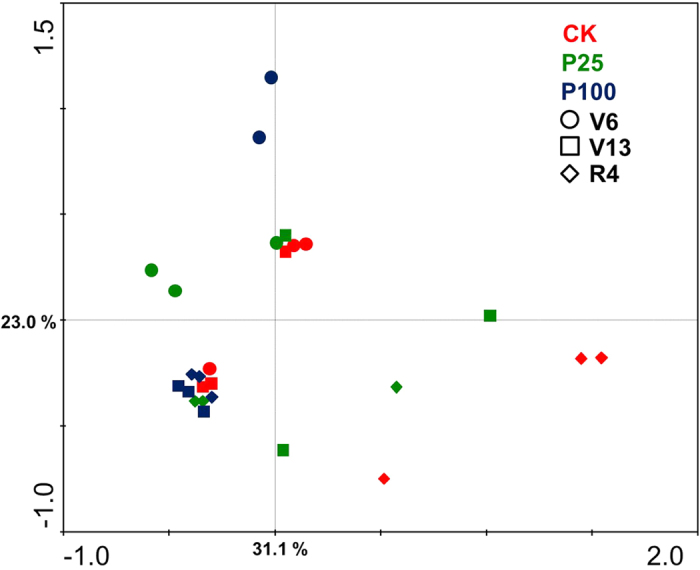
Principal component analysis (PCA) of the AM fungal community composition in the maize root in response to different growth stages. CK: zero P application (control); P25: 25 kg P ha^−1^; P100: 100 kg P ha^−1^. Solid circles represent the V6 stage, solid squares represent V13 stage, and solid diamonds represent the R4 stage. The first and second axes explain 31.1 and 23.0% of the variance.

The T-RF richness in roots increased over the growing period. P25 increased the richness at stages V13 and R4 but no significant difference was found between P0 and P100 (Fig. 1). Eleven T-RFs (97, 107, 116, 141, 157, 168, 169, 189, 190, 258, 259 bp) were detected in the maize roots (Table S2). The frequency of 97 bp and 189 bp increased in the maize roots compared with the soil (data not shown). The structure of the AMF community was significantly affected by growth stage but not by fertilizer P (Fig. 3). T-RFs 97, 116, 141, 189, 258 and 259 bp were the most frequently detected phylotypes across all maize roots over the growing period. The fractions of 116, 141 and 259 bp were detected in all root samples over the growth period. 157 bp was found at V13 and R4 stages in P25, and R4 in the control. 168 bp was detected in maize roots in the control at V13 and R4 stages but in P100 only at V6. Three T-RFs were exclusively found in one treatment or growth stage. 107 bp was detected only in the control at V6 and R4 stages. 169 bp occurred at V13 stage in P25 and 190 bp at V6 stage in P100.

### Phylogenetic analysis of AM fungi in surface soil

Three clone libraries of the AMF 18S rRNA genes were constructed from the surface soil samples (top 20 cm) in the three P treatments. Ninety-six clones were sequenced in each clone library and a neighbor-joining tree was constructed based on 70–84 effective sequences. Reference sequences were obtained from the GenBank database (Fig. S3). The rarefaction curves on the basis of analyzed sequence numbers in each clone library almost reached a plateau, indicating that the number of sequences analyzed was sufficient to characterize the AMF phylotypes present in soil and maize roots (data not shown). Twenty-seven discrete clusters were obtained from the phylogenetic analysis, and hence potentially 27 taxonomic units (each with bootstrap support >50%) were represented in the clone libraries. The 27 phylotypes are shown on the phylogenetic tree (NJ) with sequence identity from 97 to 100% and are represented by *sp1*-*sp27*. Fourteen of the 27 OTUs belonged to *Glomus*, 1 to *Funneliformis*, 3 to *Rhizophagus*, 3 to *Diversispora*, 3 to *Acaulospora*, 1 to *Sclerocystis* and 2 to *Septoglomus*. Of the effective clones sequenced from the clone library, *Glomus* (35.7–54.3%), *Funneliformis* (15.3–35.7%) *and Rhizophagus* (2.8–11.9%) were predominant, with *Acaulospora, Sclerocystis* and *Diversispora* showing lower abundance.

A combination of in silico analyses and T-RFLP fingerprinting of the representative clones shows that most of the T-RFs were assigned to Glomerales (including *Glomus* groups A and B) and five T-RFs (97, 141, 142, 169, 259 bp) were partly assigned to Diversisporales (Table S3). Of the five T-RFs, 169 bp exclusively belonged to the *Diversisporaceae*, and 142 bp exclusively belonged to the *Acaulosporaceae*, and the other T-RFs were associated with more than one sequence group. Of the T-RFs assigned to the Glomerales, 116 bp mostly belonged to *Glomus* Group A, including Rhi2 (*Rhizophagus irregularis*), Fun1 (*Funeliformis mosseae*) and Glo1 (*Glomus viscosum*). The 190 bp was mainly Glo7 (uncultured *Glomus*) and Glo9 (*Glomus* sp.). 157 bp was affiliated to *Glomus indicum* (Glo12) and 107 bp was to *Sclerocystis sinuosa* (Scl1). The T-RFs of 140 bp e and 168 bp were uncultured *Glomus* (Glo11 and Glo7 respectively). 258 bp contained mainly Glo8 and Glo10 (uncultured *Glomus*), Glo1 and Glo3 (*Glomus viscosum*).

## Discussion

Maize yields did not show any response to fertilizer P application over the three consecutive years because the soil in the present study contained relatively high levels of residual P due to previous intensive cultivation. Yet we found that mycorrhizal colonization and AMF communities were altered by P application. The fertilizer P effect started to show in the second year. Our sampling time was the tipping point in terms of soil residual P shifting from moderate P supply to P deficiency (P0). Hence, our study of the status of AMF communities at this time point is of particular interest.

Considerable evidence shows that AMF are strongly controlled by host P status and soil P availability^9^. In general, root colonization by AMF is inversely related to soil available P and plant P nutrition^25^. As expected, compared to P0, at P100 %RLC, %AC and %HC in maize roots decreased significantly, while the effect of P25 was variable and colonization sometimes increased (Table 1). At P25 soil available P at 0–20 cm depth fell within the critical P values reported for maize production (3.9 to 17.3 mg P · kg^−1^)^26^, while P100 may lead to a P-leaching risk as the value was close to 40 mg P kg^−1 ^27. Our results indicate that high P supply leads to low root infection by AMF while optimum P may possibly stimulate the potential activity of indigenous AMF in the soil. Root colonization and AM-specific Pi transporter genes are significantly up-regulated when soil Olsen-P is below a critical level (10 mg kg^−1^)^28^. The inconsistent results at P25 may be related to the heterogeneity of P distribution in the soil because fertilizer P was applied only at 0–20 cm depth but the roots were sampled to below 20 cm depth. The positive effect of P25 and the negative effect of P100 on %RLC, %AC and %HC tended to be more pronounced at R4 (Table 1), indicating that crop phenology is important in determining root colonization. High P demand and C allocation to roots at R4 in maize plants potentially affected the dependency of maize on mycorrhizal fungi to acquire P^29^. Similarly, Liu *et al*.^24^ also found changes in AMF colonization across the maize growing season and colonization increases over the growth period. In contrast to AM colonization, spore density and hyphal length density were significantly affected neither by P fertilization nor by growth stage (Table 1). These results are inconsistent with changes in spore density^30^, or the amount of AM determined by the fatty acid biomarker C16:1w5^29^ over the plant growth period. One explanation is that AM colonization is more sensitive to short-term P fertilization and crop phenology than are the growth and spore production of AMF, as the colonization structure is more closely associated with the host plant.

The influence of soil P on the diversity of AM fungi remains controversial, and the outcome is related to the forms of P fertilizer, P rates^31^, sampling times^32^ and host plant species^16^. Here, fertilizer P application did not have any overall significant effect on T-RF richness in the maize roots or in the soil (Fig. 1) except for a decrease in T-RF richness at 40–60 cm depth. Similarly, Beauregard *et al*.^32^ also found that AMF diversity was not affected by P level. By contrast, Lin *et al*.^23^ found that long-term P fertilization decreased AMF diversity and richness in an arable soil in north China.

The AMF community composition in the soil was differentiated by the fertilizer P treatment and soil depth. The separation of the AMF community among P treatments occurred at the surface soil layer and was mainly attributed to available P, N/P and pH (Fig. 2). The vertical distribution of AMF was significantly correlated with EC and Na content (Fig. 2). The impact of fertilizer P is in agreement with previous results based on agricultural soils^23^ and other ecosystems^33^. Soil pH and/or pH-driven changes in soil chemistry are important in shaping AMF communities in both natural and agricultural ecosystems^34^. The change in soil pH due to fertilizer P application might be related to plant N uptake. Phosphorus fertilization has been shown to affect N uptake and N use efficiency^35^. In addition, we found that the variation in the AMF community structure was associated with soil N/P. According to the functional equilibrium model^36^, a pronounced increase in N/P with soil depth implies that AMF deeper in the soil may enhance mutualistic benefits. However, whether this may offset the negative impact of high P on AMF in the surface soil needs further investigation. Similarly, significant effects of combined N and P fertilization on soil AMF species composition^37^ and richness^38^ have been reported. Thus, the response of the AMF community to P fertilization should also consider N levels in order to provide a more predictive picture of AM structure and function in agricultural ecosystems. The high number of T-RFs at P25 in the surface soil (0–20 cm) is particularly interesting. Whether or not this indicates that low rates of fertilizer P increase the diversity of AMF in the soil requires further study.

Of the soil physio-chemical properties determined, we found that soil EC and Na content had a significant impact on the vertical distribution of AMF in the soil. This is in accordance with our previous N fertilization study at the same site^24^ and a recent study in a semi-arid prairie ecosytem^34^. Soil salinity can impact AMF. Our study area has been substantially affected historically by high salinity and was desalinized in three stages in 1973, 1978 and 1982^39^. It is unusual that few T-RFs were detected at P100 at 40–60 cm. As the missing T-RFs (97 and 258 bp) were detected frequently in the maize roots (Table S2), this effect may be due to the spatial heterogeneity of the soil. Soil spatial heterogeneity can influence AMF communities. Alternatively, high EC and Na contents at P100 indeed act as a strong filter for specific fungal taxa. In addition to EC and Na content, it is possible that other soil properties and plant attributes also affect the vertical distribution of the AMF communities, and this requires further study.

Application of P did not have a significant influence on the community composition of AMF in the roots (Table S2). Previous studies show that some addition of fertilizer P can increase the diversity of AMF but high application rates can substantially reduce AMF diversity and change species composition^25^. By contrast, the AMF community of the three host plant species maize, viola and soybean changed significantly only at higher P concentrations (>46–70 mg l^−1^). Similarly, AMF communities associated with alfalfa were not affected by P level^32^. A large scale study in Swiss agricultural soils also reported that soil available P levels had no effect on the structure of the AMF community^40^. In the present study the soil used had a relatively high background P due to high application rates of fertilizer P to preceding crops prior to the start of the experiment. It is possible that the indigenous AMF community has been selected towards specific taxa or strains that are strong competitors and less sensitive to fertilizer P. This is supported by the six dominant representative T-RFs (97, 116, 141, 189, 258 and 259 bp) in maize roots across all P treatments and sampling times (Table S2). Hence, the residual effect of P fertilization may override the current P management practices with respect to its impacts on the AMF community, although differences in soil Olsen-P and maize P uptake were observed among the fertilizer P treatments. Nevertheless, we found that two relatively rare T-RFs (157 and 168 bp) were present only at P0 and P25 but not at P100, indicating that high application rates of fertilizer P have the potential to eliminate or reduce AMF taxa. Whether these AMF species (*Glomus indicum* and an uncultured *Glomus*) are sensitive to P fertilization requires further investigation. Evidence from cloning data shows that the relative abundance of Glo12 (affiliated with *Glomus indicum*) was substantially reduced by P fertilization (data not shown).

Seasonal variation in root AMF communities is correlated with compounding factors such as host plant species and P flux^34^, climatic conditions^41^, crop phenology^24^ and the life history traits of the fungi^42^. We found that the AMF community in maize roots shifted at certain growth stages of the maize crop. For example, R4 in the control, V13 in P25, and V6 in both P25 and P100 were separated from the remaining P treatments and growth stages. This may be due to the alteration of carbon investment in belowground parts or signaling of plants to the environment over the maize growth period. The presence of certain rare taxa (e.g. 107, 142 and 157 bp) may also affect community composition because rare species may be important in affecting AMF community structure in response to nutrient applications^38^. In the present study P fertilization did not affect the AMF community profiles in the maize roots and the AMF community was generally clustered across the different P treatments (Table S2). Likewise, the AMF community structure in the soil was altered by long-term fertilization in a wheat-rice crop rotation^43^. The frequency of occurrence of T-RFs did not differ significantly between fertilizer P treatments vs. the control (data not shown). This is consistent with findings from a long-term P fertilization (~40 years) field trial in pasture where the addition of fertilizer P to the soil was more important than the quantity applied in affecting AMF communities^33^. In the present study, N fertilizer was applied at a rate for optimal maize growth. Nitrogen fertilization at low P availability might increase the C supply belowground^36^ and enhance the colonization and diversity of AMF in maize roots in low P treatment. In addition, the common P fertilization practice in this region may have selected certain AMF taxa which show weak responses to P inputs. Future studies should include analysis of the abundance of AMF taxa in the roots to elucidate the potential mechanisms of fungal competition in the construction of AMF assemblages.

The complex linkages among climatic variation, crop phenology, soil conditions and AMF dynamics need to be clarified in long-term studies. AMF genetic diversity was found to be higher in soils than in roots in grassland ecosystems^44^. Our results are in accordance with these previous studies. We found 27 sequence types in the present study, similar to the 26 sequence types reported in maize roots in a long-term nitrogen fertilization experiment conducted in southeast Nebraska^45^ and 22 sequence types in north China^24^. Most of the sequence types were affiliated with the identified VTX reported in the Maarj*AM* database (Table S4), indicating that the VTX are ubiquitous. Six fungal T-RFs (97, 116, 141, 189, 258 and 259 bp) were detected in both maize roots and soil (Table S2). Sequence types of these OTUs belonging to the genera *Glomus, Funneliformis, Rhizophagus, Diversispora, Acaulospora, Septoglomus* and *Sclerocystis* have been frequently detected in north China^23^. The T-RFs 258 bp (*Glomus viscosum* and uncultured *Glomus*) and 97 bp (*Acaulospora mellea*, uncultured *Diversispora, Rhizophagus intraradices, Septoglomus constrictum* and *Glomus viscosum*) occurred in almost all root samples but were less frequently detected in the soil. The T-RFs 190 bp (*Glomus* sp. and uncultured *Glomus*) and 169 bp (uncultured *Diversispora*) showed the opposite trend. *Glomaceae* and *Acaulosporaceae* groups have been shown to be commonly associated with maize^43^. The enriched T-RFs indicate that maize roots showed some preference for these fungal species and selection towards certain fungal taxa. This is partly supported by the higher turnover of T-RFs in the soil than in the plant roots. Consideration of both roots and soil together provides a more comprehensive picture of AMF diversity and its potential function in agricultural ecosystems.

## Conclusions

The soil studied had relatively high residual P levels and soil available P showed some response to P supply but maize yields showed no response after three years of fertilizer P application. The responses of AMF communities in maize roots and soil to fertilizer P application are complex. Here, we found that P fertilization significantly affected root colonization but not the diversity or community structure of AMF in maize roots. The temporal changes in the AMF community in maize roots indicate that crop phenology might override fertilizer P in determining the community composition of active root inhabiting fungi. By contrast, a shift in AMF community seen in the surface soil is mainly attributable to soil available P and pH, and optimum P tends to increase the diversity of AMF. The vertical distribution of AMF in the soil is related to soil EC and Na content. Hence, P management should be integrated with cropping design and other agricultural practices to ensure the sustainable agricultural production in these salinized soils.

## Materials and Methods

### Study site description and experimental design

The study site was a fertilizer P experiment site which was established in 2008. The site is located at China Agricultural University’s Quzhou Experimental station, Quzhou County, Hebei province, China (36°52′N, 115°02′E). The silt fluvo-aquic soil contained 0.67 g kg^−1^ total N, Olsen-P 7 mg kg^−1^, exchangeable-K 74 mg kg^−1^, organic matter content 10.3 g kg^−1^; and had a pH of 8.5 (1:2.5 soil/water w/v) in the top 30 cm before planting in 2008^46^. The climate is warm and sub-humid with an average annual temperature of 13.2 °C and precipitation ranging from 213 to 840 mm. The cropping system is a winter wheat-summer maize rotation in each year and is representative of the typical cropping system on the North China Plain. Winter wheat (*Triticum aestivum* L. cv. Kenong9204 and Shijiazhuang8) was planted in mid-October and harvested in mid-June and summer maize (*Zea mays* L. cv. Nongda 108 and NE 15) was sown in mid-June and harvested in mid-October. Mean yields of wheat and maize in this region are 6.5 and 8.0 t ha^−1^, respectively.

The design of the experiment was a randomized complete block with four replicates. The size of each plot was 5.4 m × 8 m. Two maize cultivars (*Zea mays* L. cv. Nongda 108 and Ne15) were sown but only NE15 was sampled. Planting density of maize was approximately 67500 individual seeds per hectare with a 60 cm row width. The total experiment consisted six application rates of fertilizer P (0, 12.5, 25, 50, 100 and 200 kg P ha^−1^) but only three P application rates were chosen for this study, namely Control (no fertilizer P input), P25 (25 kg P ha^−1^) and P100 (100 kg P ha^−1^). Before sowing, straw was removed from the field and the entire application of P (as calcium superphosphate), 75 kg N ha^−1^ (as urea), and 50 kg K ha^−1^ (as potassium sulfate) were broadcast and mixed with the surface soil by disking. At the 13-leaf stage an additional 150 kg N ha^−1^ as urea was top-dressed to each plot. Irrigation, herbicides and pesticides were used according to the local practice when necessary. Grain yield was determined by manually harvesting and drying (at 60 °C) ears from two rows per plot.

### Soil and plant sampling

Soil was sampled in October 2011 after the maize harvest, i.e. three years after the establishment of the experiment. Soil samples were collected from different depths (0–20, 20–40 and 40–60 cm) using a soil core sampler (3 cm internal diameter). Each plot was divided to four quadrants, and sampling was conducted in the middle of each quadrant. Hence four soil cores were collected at each plot and mixed to give one composite sample at each depth. A total of 36 mixed stratified soil samples were collected. The samples were sieved (<2 mm) and divided into two portions. One was stored at −20 °C for DNA extraction and subsequent molecular analysis and the other portion was air-dried and used for analysis of soil physico-chemical properties.

Maize root samples were taken at growth stages V6 (20 July, 6-leaf collar), V13 (5 August, 13-leaf collar), and R4 (19 September, kernel dough). Three maize plants were sampled along a transect at three sampling points at 1-m intervals. Shoots and roots were separated and rhizosphere soil was collected using the shaking method. Roots of three maize plants were mixed to form one composite sample from each plot. Root samples were divided into two portions, one of which was stored at −20 °C for DNA extraction and the other retained for determination of percentage root length colonized by AMF.

### Soil and plant physico-chemical properties

Soil pH was determined by glass electrode (1:2 soil/water, w/v). Soil electrical conductivity (EC) was measured with an electrical conductivity meter. Soil total N was determined by the Dumas combustion method (Elemental Analyzer Vario EI III, Elementar Analysensysteme GmbH, Hanau, Germany). Other soil properties determined were soil organic matter (soil digestion with hot acid dichromate^47^) and available P (Olsen-P, 0.5 M NaHCO_3_^48^). Soil Na and Zn concentrations were determined by inductively coupled plasma optical emission spectroscopy (ICP-AES, OPTIMA 3300 DV, Perkin-Elmer, Waltham, MA) after Mehlich 3 solution extraction^49^. Soil N/P ratio was calculated based on total N and available P. Plant shoots were over-dried at 60 °C for three days, weighed and ground for nutrient analysis. Plant P concentration was measured by the molybdo-vanadophosphate method after samples were digested with concentrated H_2_SO_4_ and H_2_O_2_^28^.

### Assessment of AM fungal colonization, spore density and hyphal length density

Fine roots were rinsed with distilled water and cleared with 10% KOH. Cleared root samples were thoroughly rinsed with distilled water and stained with 0.05% (w/v) Trypan blue^50^. A random subset of thirty 1-cm-long root segments from each sample was mounted onto microscope slides. The percentage root length colonized by Glomeromycota was quantified using the magnified intersection method with 200 intersections^51^ and some other mycorrhizal indicators (root length colonization, %RLC; arbuscular colonization, %AC; hyphal colonization, %HC) were also determined. Spores of AMF in the rhizosphere soil (2-mm sieved) were counted using the method described by Daniels and Skipper^52^. Ten grams of soil were taken from each soil sample and wet-sieved. AMF spores were counted on a grid pattern dish under a binocular stereomicroscope. Hyphal length density was determined according to Jakobsen *et al*.^53^.

### DNA extraction and amplification

Soil DNA was extracted from 0.5 g fresh soil using a FastDNA Spin Kit for Soil (Bio101, Carlsbad, CA) and maize root genomic DNA was extracted from 0.05 g frozen root samples (ground and homogenized with liquid nitrogen) using a Fast Plant Kit (Tiangen, Beijing, China) following the manufacturers’ instructions. The total DNA concentration in each soil or root sample was quantified spectrophotometrically using a NanoDrop ND-8000 (NanoDrop, Wilmington, DE). AM fungal specific 5′-labeled primer pairs NS31-HEX and AM1-FAM^54^ were used to amplify the AMF SSU rDNA gene (~550 bp) in soil and root sample extracts for subsequent T-RFLP analysis and the conventional NS31/AM1 primer pair (without fluorescence labeling) was used for subsequent clone-sequencing analysis^55^. Because polysaccharide components in roots would interfere with the PCR amplification and one step PCR was difficult to amplify satisfactory root products, the fungal primer pair AML1/AML2^56^ was used prior to the amplification of AM fungal specific primer. A 10 μl PCR reaction system contained 5 μl 2× TaqMix (Tiangen Co., Ltd, Beijing, China), 0.2 μl 10 mM each primer, 1 μl DNA template (about 100 ng of soil or root extracted template DNA) and 3.6 μl ddH_2_O. Thermal cycling for soil samples was performed as described by Helgason *et al*.^57^ with some modification: 5 min initial denaturation at 94 °C; 40 cycles of 30 s denaturation at 94 °C, 1 min annealing at 58 °C and 1 min elongation at 72 °C; and a 10 min final elongation at 72 °C. The cycling parameters for root samples consisted of an initial denaturing step for 5 min at 94 °C, 34 (round one) or 30 (round two) cycles consisting of 30 s at 94 °C, 45 s at 60 °C and 1 min at 72 °C, followed by a final extension step of 72 °C for 10 min. 5 μL PCR products were checked on 1% agarose gel to estimate the quantity of PCR products. The PCR product with the strongest band on the gel and the expected targeted fragment length of AMF (~550 bp) was selected and purified with the QIAquick PCR Purification Kit (Tiangen, Beijing, China).

### T-RFLP analysis

PCR products were digested with the restriction enzymes Hinfl and Hin1II (both Promega) in separate reactions^54^. The 10 μl digestion reaction consisted of 3.0 μl diluted (1:5) PCR product and 1 unit Hinfl or Hin1II in the buffer recommended by the manufacturers. The reactions were incubated at 37 °C for 3 h followed by a heat inactivation step at 80 °C for 20 min. Samples were purified with Exo-SAP-IT (USB, Cleveland, OH) followed by a Sephadex column clean-up step and mixed with formamide and the internal lane size standard ROX-500-ILS (Microread). The size of the terminal restriction fragments (T-RFs) in each sample was determined using an ABI-PRISM 3130XL Genetic Analyzer (Applied Biosystems, Carlsbad, CA) by Yuewei Gene Company (Beijing, China). Fragment data were analyzed using Peak Scanner software v1.0 (Applied Biosystems). Only T-RFs ranging from 45 to 450 base pairs in length with a minimum peak height of 50 relative fluorescent units and accounting for 1% of the total peak profile for each sample were considered for further analysis^58^. Two types of fluorescence label (HEX and FAM) and two restriction enzymes (Hinf1 and Hin1II) were used^54^. However, the two restriction enzymes failed to digest T-RFs labeled by FAM. Thus, only HEX labeled T-RFs were used for subsequent analysis.

The automated sequencer detects all fluorescent DNA fragments. If there is a strong secondary structure or partial digestion, a signal that does not correspond to a true T-RF can be detected. T-RFLP requires matching unknown T-RFLP profiles to a database of known T-RFLP patterns to identify which species or taxa are in a sample. In the present experiment we used the clone-sequencing method to construct an AMF T-RFLP profile database.

### Cloning, sequencing and clone library construction

Our cloned region was part of the SSU rDNA gene (length ~550 bp). Surface soil samples (0–20 cm depth) were used for AMF T-RFLP profile database construction. Primer pair (without fluorescence labeling) and PCR cycling parameters of the clone-sequencing procedure were similar to those described above. Twelve soil samples (3 P treatments, 4 replicates) were amplified with NS31/AM1 and the 4 replicated PCR products of each P treatment were pooled together to form one sample for clone library construction. In this study three soil sample clone libraries were built to distinguish the identification of T-RFs.

Cloning was conducted by the method of Liu *et al*.^24^ and Lee *et al*.^56^. In each clone library, clones containing the converted DNA fragments were selected by blue/white screening and 96 white clones were randomly picked up. All clones were sequenced by ZhongKeXiLin Biotechnology Company (ABI 3730XL, Beijing, China). After sequencing, each positive clone was used as a template for PCR amplification (NS31-HEX/AM1-FAM) and was digested and T-RFLP analysis was performed. AMF sequences obtained from the clone library were also used in simulated restriction endonuclease enzyme digestion using ChromasPro software using Hinfl and Hin1II. Unknown T-RFLP profiles were matched with profiles of sequenced clones and all T-RFs within 1.5 base pairs were required to be detected for a positive match. T-RFLP detected all or almost all terminal restriction fragments predicted from the cloned sequences. This step allowed us to conclude with confidence that all peaks taken into account were true T-RFs even when they were of low intensity.

### Phylogenetic analysis

The size of the sequence amplified by this primer pair was about 550 bp and sequences were examined with BLAST^59^ to determine whether sequences were derived from Glomeromycota (http://www.ncbi.nlm.nih.gov/) and searched for closely related sequences. All sequences were grouped into operational taxonomic units (OTUs) using the DOTUR-1.53 program with similarities >97%. For the phylogenetic analyses on sequence data, the closest matches to each OTU were determined using the BLAST sequence similarity search tool against GenBank^59^ and retained as references. The alignment was conducted by ClustalX and MEGA 4 was used to construct the phylogenetic tree, and the phylogenetic tree was inferred by the neighbor joining (NJ) method using Kimura 2-parameter with a bootstrap support of >80% (1000 re-samplings). *Endogone pisiformis* (X58724) was used as outer group. Distance matrices were constructed by the DNADIST program in PHYLIP for sequence data. The AM fungal sequences used in phylogenetic analysis were submitted to the GenBank database under the accession numbers KT291187-KT291412. In addition, our sequences were blasted against the online database Maarj*AM*^60^ (http://maarjam.botany.ut.ee/; status on July 18, 2015) and grouped into the corresponding molecular virtual taxa with the sequence identity ≥97%. The numbers of publications reporting the same sequence types are listed in Table S4.

### Data analysis and statistics

The T-RFs which matched with sequences in the clone library in each P treatment were defined as valid T-RFs. The T-RF matrix in each sample was constructed by the presence/absence of individual T-RF. T-RF richness (S) was represented by the total number of T-RFs in soil or maize roots at three growth stages under different P treatments. Significant differences among soil physico-chemical properties, infection indexes and diversity indexes of AMF were tested by one-way analysis of variance followed by comparison between pairs of mean values using Duncan’s multiple range test. Two-way analysis of variance was used to analyze the main and interactive effects of P fertilization and soil depth on each soil chemical property and AMF infection index. We assessed relationships between richness and soil chemical variables by calculating the Pearson’s correlation coefficients. All statistical analyses were performed using SPSS 13.0 (SPSS Inc., Chicago, IL). Canonical correspondence analysis (CCA) was performed to analyze the influence of P fertilization treatments and soil physico-chemical properties on soil AMF community composition. Principal component analysis (PCA) was explored to analyze the influence of P fertilization and maize growth stage on root AMF community structure. Ordination analyses and hypothesis testing were conducted in CANOCO for Windows v. 4.5 with binary-transformed data. In addition, forward selection tests were conducted using 499 permutations and the Monte Carlo permutation test with *p* < 0.05 was used. Biplots were created using CanoDraw 4.5 to display the ordination results.

## Additional Information

**How to cite this article**: Liu, W. *et al*. Arbuscular mycorrhizal fungi in soil and roots respond differently to phosphorus inputs in an intensively managed calcareous agricultural soil. *Sci. Rep.* **6**, 24902; doi: 10.1038/srep24902 (2016).

## Supplementary Material

Supplementary Information

## Figures and Tables

**Table 1:** Percentage root colonization, spore density, and hyphal length density in different P treatments and at different growth stages.

Growth stage	Treatment	Root length colonization (%RLC)	Arbuscular colonization (%AC)	Hyphal colonization (%HC)	Hyphal length density (m g^−1^)	Spore density (g^−1^ soil)
V6	Control	29.04 ± 2.53b	8.24 ± 3.22cd	12.65 ± 2.84bc	1.23 ± 0.12bc	7.21 ± 1.10ab
	P25	16.40 ± 3.99bc	0.86 ± 0.47d	5.66 ± 2.17c	1.77 ± 0.16a	6.38 ± 0.41abc
	P100	11.10 ± 1.43c	0.80 ± 0.52d	3.06 ± 0.75c	1.48 ± 0.09abc	6.16 ± 0.98abc
V13	Control	54.23 ± 7.49a	20.44 ± 4.73ab	32.92 ± 9.72a	1.45 ± 0.16abc	7.93 ± 1.22a
	P25	46.85 ± 2.70a	13.46 ± 1.80bc	25.24 ± 1.47ab	1.29 ± 0.25abc	6.22 ± 0.77abc
	P100	42.59 ± 6.04a	15.86 ± 3.77bc	26.64 ± 6.49ab	1.15 ± 0.07c	7.28 ± 0.77ab
R4	Control	43.56 ± 1.70a	18.63 ± 1.73b	22.93 ± 1.55ab	1.32 ± 0.05abc	5.78 ± 0.72abc
	P25	53.56 ± 1.80a	28.33 ± 2.13a	29.10 ± 0.62a	1.67 ± 0.22ab	4.73 ± 0.45bc
	P100	23.95 ± 2.75bc	8.60 ± 1.85cd	12.47 ± 2.42bc	1.29 ± 0.13abc	3.86 ± 0.25c
Analysis of variance						
P fertilization level		**	*	ns	ns	ns
Growth stage		***	***	***	ns	**
P fertilization level × Growth stage		*	**	ns	ns	ns

Data are mean values ± SE (n = 4). Significant differences among P treatments and growth stage within each variable were tested using Duncan’s multiple range test (*p* < 0.05) and are indicated by different letters. Two-way ANOVA was used to represent the main and interactive effects of P treatments and growth stage on each mycorrhizal parameter. Control: zero fertilizer P; P25: low-P treatment; P100: high-P treatment. V6, V13 and R4 refer to 6-leaf collar, 13-leaf collar and kernel dough during maize growth period. *p < 0.05; **p < 0.01; ***p < 0.001; ns, not significant.

## References

[b1] Schachtman D. P., Reid R. J. & Ayling S. M. Phosphorus uptake by plants: from soil to cell. Plant Physiol. 116, 447–453 (1998). 9490752 10.1104/pp.116.2.447 PMC1539172

[b2] Zhang W. . Efficiency, economics, and environmental implications of phosphorus resource use and the fertilizer industry in China. Nutr. Cycl. Agroecosyst. 80, 131–144 (2008).

[b3] Zhang W. L., Xu A. G., Ji H. J. & Kolbe H. Estimation of agricultural non-point source pollution in chinaand the alleviating strategies III. A review of policies and practices for agricultural non-point source pollution control in China. Scientia Agric. Sinica 37, 1008–1017 (2004).

[b4] Bai Z. . Erratum to: The critical soil P levels for crop yield, soil fertility and environmental safety in different soil types. Plant Soil 372, 39-39 (2013).

[b5] Li H. . Past, present, and future use of phosphorus in Chinese agriculture and its influence on phosphorus losses.Ambio 44, 274–285 (2015). 10.1007/s13280-015-0633-0 PMC4329154 25681984

[b6] Kirkby E. A. & Johnston A. E. Soil and fertilizer phosphorus in relation to crop nutrition in The ecophysiology of plant-phosphorus interactions (eds White P. J. & Hammond J. P.) Ch. 9, 177–223 (Springer: Netherlands,, 2008).

[b7] Shen J. B. . Maximizing root/rhizosphere efficiency to improve crop productivity and nutrient use efficiency in intensive agriculture of China. J. Exp. Bot. 64, 1181–1192 (2013). 23255279 10.1093/jxb/ers342

[b8] Richardson A. E. Soil microorganisms and phosphorus availability in Soil Biota: Management in Sustainable Farming Systems (eds Pankhurst C. E. .) 50–62 (Mclbournc, Australia: CSIRO, 1994).

[b9] Smith S. E. & Read D. J. in Mycorrhizal Symbiosis 3rd edn (eds Smith S. E. & Read D. J.) 13–41 (Academic Press, Cambridge, UK, 2008).

[b10] Smith S. E. & Smith F. A. Fresh perspectives on the roles of arbuscular mycorrhizal fungi in plant nutrition and growth. Mycologia 104, 1–13 (2012). 21933929 10.3852/11-229

[b11] Verbruggen E., van der Heijden M. G. A., Rillig M. C. & Kiers E. T. Mycorrhizal fungal establishment in agricultural soils: factors determining inoculation success. New Phytol. 197, 1104–1109 (2013). 23495389 10.1111/j.1469-8137.2012.04348.x

[b12] Jakobsen I. Transport of phosphorus and carbon in VA mycorrhizas in Mycorrhiza (eds Varma A. & Hock B.) 297–324 (Springer, Germany, 1995).

[b13] Olsson P. A., Bååth E. & Jakobsen I. Phosphorus effects on the mycelium and storage structures of an arbuscular mycorrhizal fungus as studied in the soil and roots by analysis of fatty acid signatures. Appl. Environ. Microbiol. 63, 3531–3538 (1997). 16535691 10.1128/aem.63.9.3531-3538.1997 PMC1389247

[b14] Gosling P., Hodge A., Goodlass G. & Bending G. D. Arbuscular mycorrhizal fungi and organic farming. Agr. Ecosyst. Environ. 113, 17–35 (2006).

[b15] Beauregard M. S. . Various forms of organic and inorganic P fertilizers did not negatively affect soil- and root-inhabiting am fungi in a maize–soybean rotation system. Mycorrhiza 23, 143–154 (2013). 22961069 10.1007/s00572-012-0459-6

[b16] Gosling P., Andrew M., Maude P., Hammond J. P. & Bending G. D. Contrasting arbuscular mycorrhizal communities colonizing different host plants show a similar response to a soil phosphorus concentration gradient. New Phytol. 198, 546–556 (2013). 23421495 10.1111/nph.12169 PMC3798118

[b17] Harikumar V. S. Arbuscular mycorrhizal associations in sesame under low-input cropping systems. Arch. Agron. Soil Sci. 61, 347–359 (2015).

[b18] Oehl F., Sieverding E., Ineichen K., Elisabeth-Anne R., Boller T. & Wiemken A. Community structure of arbuscular mycorrhizal fungi at different soil depths in extensively and intensively managed agroecosystems. New Phytol. 165, 273–283 (2005). 15720639 10.1111/j.1469-8137.2004.01235.x

[b19] Li X. . Inner Mongolian steppe arbuscular mycorrhizal fungal communities respond more strongly to water availability than to nitrogen fertilization. Environ. Microbiol. 17, 3051–3068 (2015). 26033305 10.1111/1462-2920.12931

[b20] Cao N., Chen X., Cui Z. & Zhang F. Change in soil available phosphorus in relation to the phosphorus budget in China. Nutr. Cycl. Agroecosyst. 94, 161–170 (2012).

[b21] CAY. in China Agriculture Yearbook (ed. CAY) (Chinese Agricultural Press, Beijing, 2009).

[b22] Vitousek P. M. . Nutrient imbalances in agricultural development. Science 324, 1519–1520 (2009). 19541981 10.1126/science.1170261

[b23] Lin X. . Long-term balanced fertilization decreases arbuscular mycorrhizal fungal diversity in an arable soil in north china revealed by 454 pyrosequencing. Environ. Sci. Technol. 46, 5764–5771 (2012). 22582875 10.1021/es3001695

[b24] Liu W. . Spatiotemporal changes in arbuscular mycorrhizal fungal communities under different nitrogen inputs over a 5-year period in intensive agricultural ecosystems on the North China Plain. FEMS Microbiol. Ecol. 90, 436–453 (2014). 25098725 10.1111/1574-6941.12405

[b25] Kahiluoto H., Ketoja E., Vestberg M. & Saarela I. Promotion of AM utilization through reduced P fertilization 2. Field studies. Plant Soil 231, 65–79 (2001).

[b26] Xu T. . Determining critical values of soil Olsen-P for maize and winter wheat from long-term experiments in china. Plant Soil 323, 143–151 (2009).

[b27] Zhong X. . The evaluation of phosphorus leaching risk of 23 Chinese soils I. Leaching criterion. Acta Ecol. Sin. 24, 2275–2280 (2004).

[b28] Deng Y. . Is the inherent potential of maize roots efficient for soil phosphorus acquisition. PLos One 9, e90287 (2014). 24594677 10.1371/journal.pone.0090287 PMC3940875

[b29] Tian H., Drijber R. A., Niu X. S., Zhang J. L. & Li X. L. Spatio-temporal dynamics of an indigenous arbuscular mycorrhizal fungal community in an intensively managed maize agroecosystem in north china. Appl. Soil Ecol. 47, 141–152 (2011).

[b30] Bhadalung N. N. . Effects of long-term np-fertilization on abundance and diversity of arbuscular mycorrhizal fungi under a maize cropping system. Plant Soil 270, 371–382 (2005).

[b31] Mathimaran N., Ruh R., Vullioud P., Frossard E. & Jansa J. *Glomus intraradices* dominates arbuscular mycorrhizal communities in a heavy textured agricultural soil. Mycorrhiza 16, 61–66 (2005). 16133255 10.1007/s00572-005-0014-9

[b32] Beauregard M. S., Hamel C., Atul-Nayyar & St-Arnaud M. Long-term phosphorus fertilization impacts soil fungal and bacterial diversity but not AM fungal community in alfalfa. Microbial. Ecol. 59, 379–389 (2009). 10.1007/s00248-009-9583-z 19756847

[b33] Wakelin S. . Response of soil microbial communities to contrasted histories of phosphorus fertilization in pastures. Appl. Soil Ecol. 61, 40–48 (2012).

[b34] Bainard L. D., Bainard J. D., Hamel C. & Gan Y. Spatial and temporal structuring of arbuscular mycorrhizal communities is differentially influenced by abiotic factors and host crop in a semi-arid prairie agroecosystem. FEMS Microbiol. Ecol. 88, 333–344 (2014). 24527842 10.1111/1574-6941.12300

[b35] Yang C. . Management of the Arbuscular Mycorrhizal Symbiosis in Sustainable Crop Production in Mycorrhizal Fungi: Use in Sustainable Agriculture and Land Restoration (eds Solaiman Z. M. .) 89–119 (Springer Press, Heidelberg, 2014).

[b36] Johnson N. C. Resource stoichiometry elucidates the structure and function of arbuscular mycorrhizas across scales. New Phytol. 185, 631–647 (2009). 19968797 10.1111/j.1469-8137.2009.03110.x

[b37] Chen Y. L. . Six-year fertilization modifies the biodiversity of arbuscular mycorrhizal fungi in a temperate steppe in Inner Mongolia. Soil Biol. Biochem. 69, 371–381 (2014).

[b38] Camenzind T. . Nitrogen and phosphorus additions impact arbuscular mycorrhizal abundance and molecular diversity in a tropical montane forest. Global Change Biol. 20, 3646–3659 (2014). 10.1111/gcb.12618 24764217

[b39] Liu Y., Yu Z., Gu W. & Axmacher J. C. Diversity of carabids (Coleoptera, Carabidae) in the desalinized agricultural landscape of Quzhou County, China. Agric. Ecosyst. Environ. 113, 45–50 (2006).

[b40] Jansa J., Erb A., Oberholzer H., Šmilauer P. & Egli S. Soil and geography are more important determinants of indigenous arbuscular mycorrhizal communities than management practices in Swiss agricultural soils. Mol. Ecol. 23, 2118–2135 (2014). 24611988 10.1111/mec.12706

[b41] Vicca S. . Arbuscular mycorrhizal fungi may mitigate the influence of a joint rise of temperature and atmospheric CO_2_ on soil respiration in grasslands. Int. J. Ecol. 2009, 10 (2009).

[b42] Oehl F. . Revision of Glomeromycetes with Entrophosporoid and Glomoid spore formation with three new genera. Mycotaxon 117, 297–316 (2011).

[b43] Qin H. . Long-term fertilizer application effects on the soil, root arbuscular mycorrhizal fungi and community composition in rotation agriculture. Appl. Soil Ecol. 89, 35–43 (2015).

[b44] Hempel S., Renker C. & Buscot F. Differences in the species composition of arbuscular mycorrhizal fungi in spore, root and soil communities in a grassland ecosystem. Environ. Microbiol. 9, 1930–1938 (2007). 17635540 10.1111/j.1462-2920.2007.01309.x

[b45] Tian H., Drijber R. A., Zhang J. L. & Li X. L. Impact of long-term nitrogen fertilization and rotation with soybean on the diversity and phosphorus metabolism of indigenous arbuscular mycorrhizal fungi within the roots of maize (*Zea mays* L.). Agric. Ecosyst. Environ. 164, 53–61 (2013).

[b46] Meng Q. F. . Alternative cropping systems for sustainable water and nitrogen. Agric. Ecosyst. Environ. 146, 93–102 (2012).

[b47] Bremner J. M. Part 3-chemical methods in Methods of Soil Analysis (eds Sparks D. L. .) 1085–1121 (ASA and SSSA, Madison, 1996).

[b48] Olsen S. R., Cole C. V., Watanabe F. S. & Dean L. A. Estimation of available phosphorus in soils by extraction with sodium bicarbonate in Miscellaneous paper institute for agricultural research samara Pp (USDA, Washington DC, 1954).

[b49] Mehlich A. Mehlich 3 soil test extractant: a modification of Mehlich 2 extractant. Commun. Soil Sci. Plant. Anal. 15, 1409–1416 (1984).

[b50] Phillips J. M. & Hayman D. S. Improved procedures for clearing roots and staining parasitic and vesicular arbuscular mycorrhizal fungi for rapid assessment of infection. Trans. Brit. Mycol. Soc. 55, 158–161 (1970).

[b51] McGonigle T. P., Miller M. H., Evans D. G., Fairchild G. L. & Swan J. A. A new method which gives an objective measure of colonization of roots by vesicular-arbuscular mycorrhizal fungi. New Phytol. 115, 495–501 (1990). 10.1111/j.1469-8137.1990.tb00476.x 33874272

[b52] Daniels B. A. & Skipper H. D. Methods for the recovery and quantitative estimation of propagules from soil in Methods and Principles of Mycorrhizal Research (ed. Schenck N. C.) 29–35 (American Phytopathological Society, St. Paul, 1982).

[b53] Jakobsen I., Abbott L. K. & Robson A. D. External hyphae of vesicular arbuscular mycorrhizal fungi associated with *Trifolium subterraneum* L. 1. Spread of hyphae and phosphorus inflow into roots. New Phytol. 120, 371–379 (1992).

[b54] Dickie I. A. & FitzJohn R. G. Using terminal restriction fragment length polymorphism (T-RFLP) to identify mycorrhizal fungi: a methods review. Mycorrhiza 17, 259–270 (2007). 17429700 10.1007/s00572-007-0129-2

[b55] Helgason T., Daniell T. J., Husband R., Fitter A. H. & Young J. P. W. Ploughing up the wood-wide web? Nature 394, 431 (1998). 9697763 10.1038/28764

[b56] Lee J., Lee S. & Young J. P. W. Improved PCR primers for the detection and identification of arbuscular mycorrhizal fungi. FEMS Microbiol. Ecol. 65, 339–349 (2008). 18631176 10.1111/j.1574-6941.2008.00531.x

[b57] Helgason T., Fitter A. H. & Young J. P. W. Molecular diversity of arbuscular mycorrhizal fungi colonising *Hyacinthoides non-scripta* (bluebell) in a semi-natural woodland. Mol. Ecol. 8, 659–666 (1999).

[b58] Johnson N. C., Rowland D., Corkidi L., Egerton-Warburton L. M. & Allen E. B. Nitrogen enrichment alters mycorrhizal allocation at five mesic to semiarid grasslands. Ecology 84, 1895–1908 (2003).

[b59] Altschul S. F. . Gapped BLAST and PSI-BLAST: a new generation of protein database search programs. Nucl. Acid. Res. 25, 3389–3402 (1997). 10.1093/nar/25.17.3389 PMC146917 9254694

[b60] Öpik M. . The online database Maarj*AM* reveals global and ecosystemic distribution patterns in arbuscular mycorrhizal fungi (Glomeromycota). New Phytol. 188, 223–242 (2010). 20561207 10.1111/j.1469-8137.2010.03334.x

