# Supplementary material for: Arbuscular mycorrhizal fungi in soil and roots respond differently to phosphorus inputs in an intensively managed calcareous agricultural soil
Source: Sci Rep. 2016 Apr 22;6:24902. doi: 10.1038/srep24902 (PMC4840358; doi:10.1038/srep24902)
Supplement: Supplementary Information [file srep24902-s1.doc]

**Arbuscular mycorrhizal fungi in soil and roots respond differently to phosphorus inputs in an intensively managed calcareous agricultural soil**

Wei Liu, Yunlong Zhang, Shanshan Jiang, Yan Deng, Peter Christie, Philip J. Murray, Xiaolin Li & Junling Zhang

**Supplementary Information (Tables and Figures)**

**Figure S1** Rank-frequency of the fungal T-RFs profiles in the soil. The y-axis shows the occurrence of each T-RF detected in soil samples and the x-axis is the ordinal rank of the T-RFs from the most frequent to the least frequent.

**Figure S2** Relationship between frequency of occurrence of taxa in roots and in soil. Gray circles represent occurrence of taxa in both soil and roots; black circles represent taxa occurring only in soil.

**Figure S3** Neighbor-joining phylogenetic tree of representative sequences of each phylotype of AM fungi obtained from 0-20 cm soil depth and the referenced sequences from GenBank. Bootstrap values (1000 replicates) are shown. Sequence groups were delimited according to the sequence similarity at the 97 % level.

**Table S1** Selected soil physico-chemical properties under different P treatments.

**Table S2** AM fungal phylotypes detected from soil and root samples of different maize growth stages under different P treatments.

**Table S3** Characterization of T-RFs to phylogenetic lineage in the NJ phylogenetic tree of representative sequences. The numbers indicate sequence numbers recovered in the clone library which are affiliated with each T-RF.

**Table S4** Molecular virtual taxon and related morphospecies in the Maarj*AM* database (http://maarjam.botany.ut.ee/, Öpik et al. 2010) corresponding with OTUs detected in the present study. Only OTUs with ≥ 97% maximum identity were included with molecular virtual taxon. Numbers in the table show the publications found in the *MaarjAM* database.


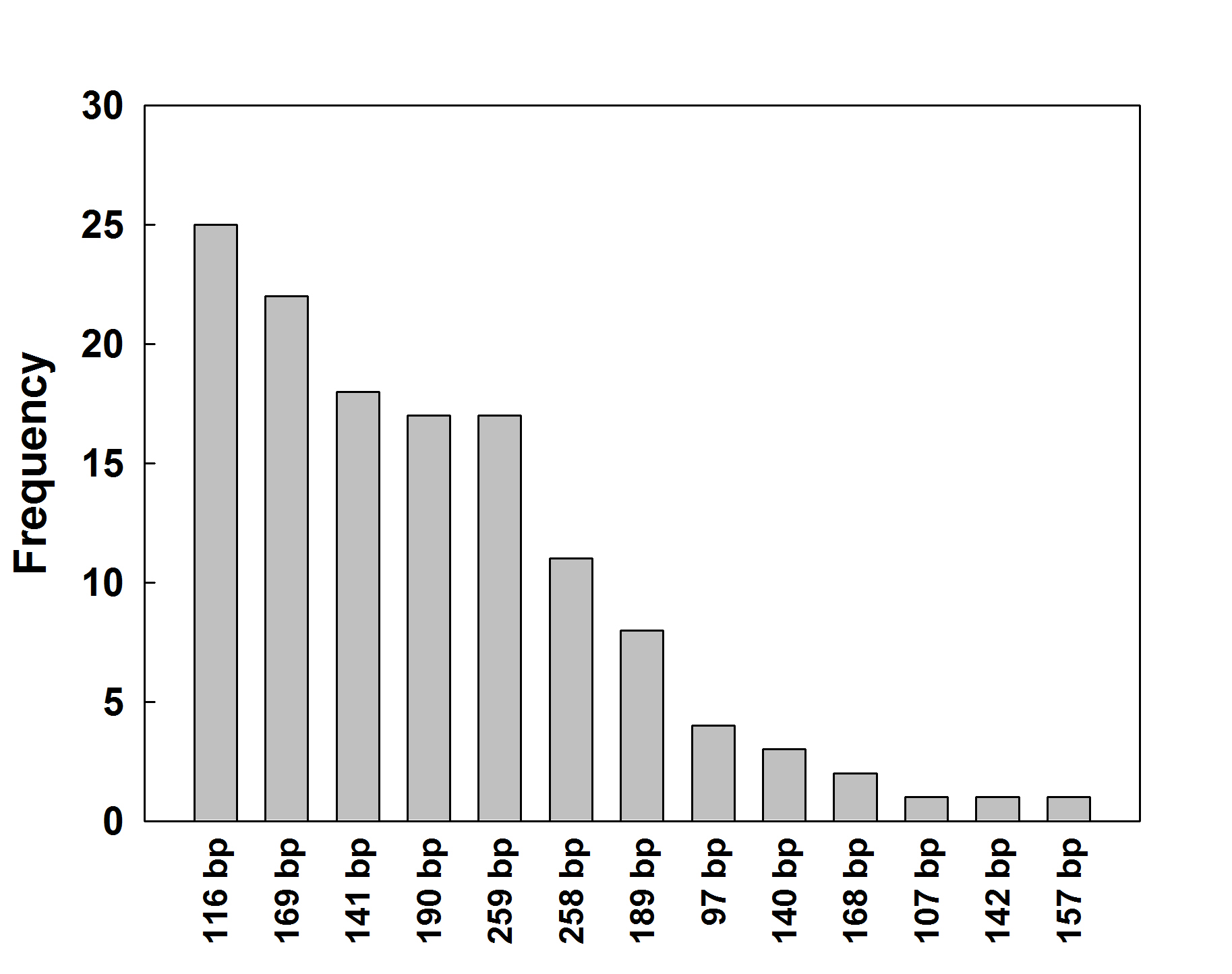


**Figure S1**

**
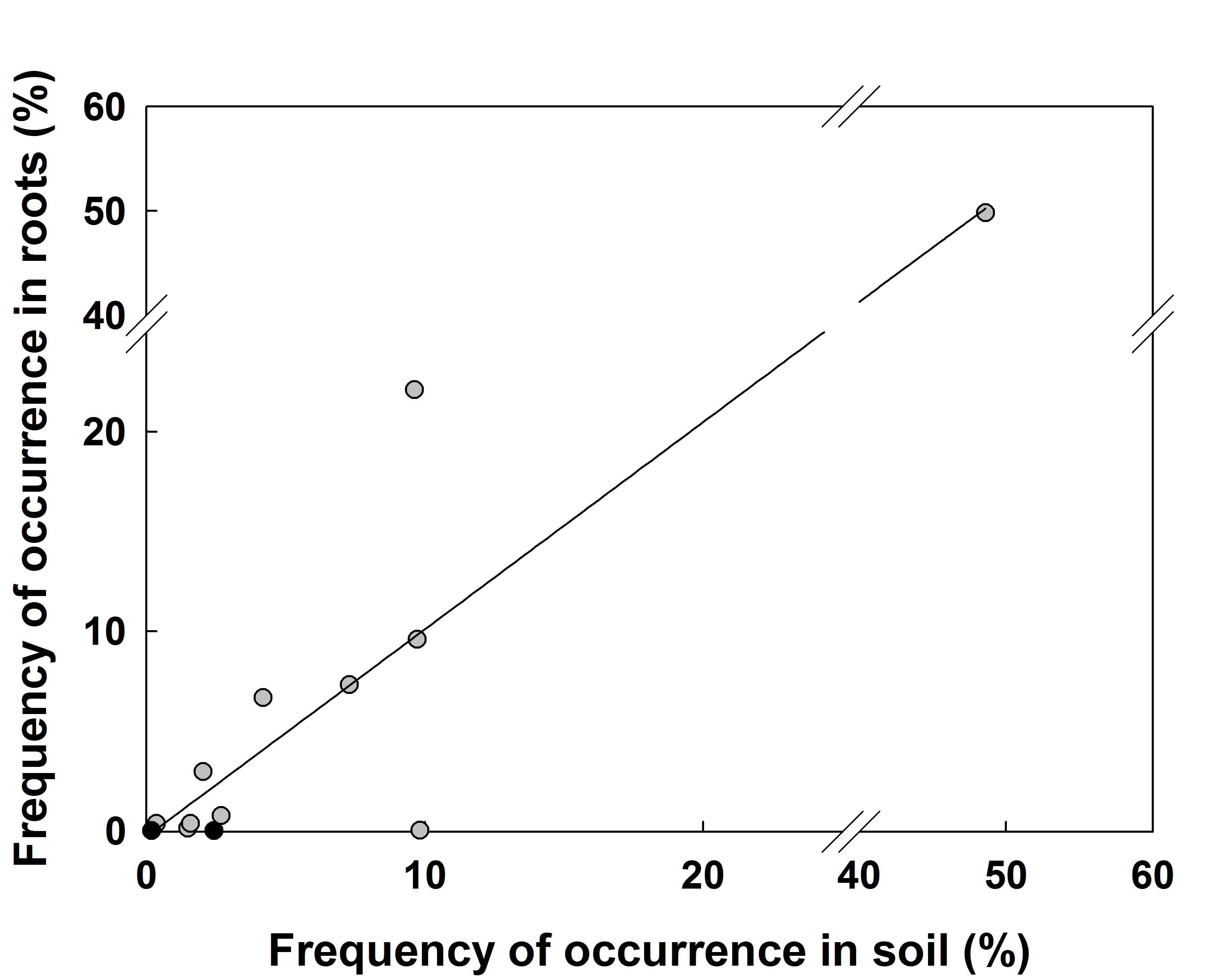
**

**Figure S2**


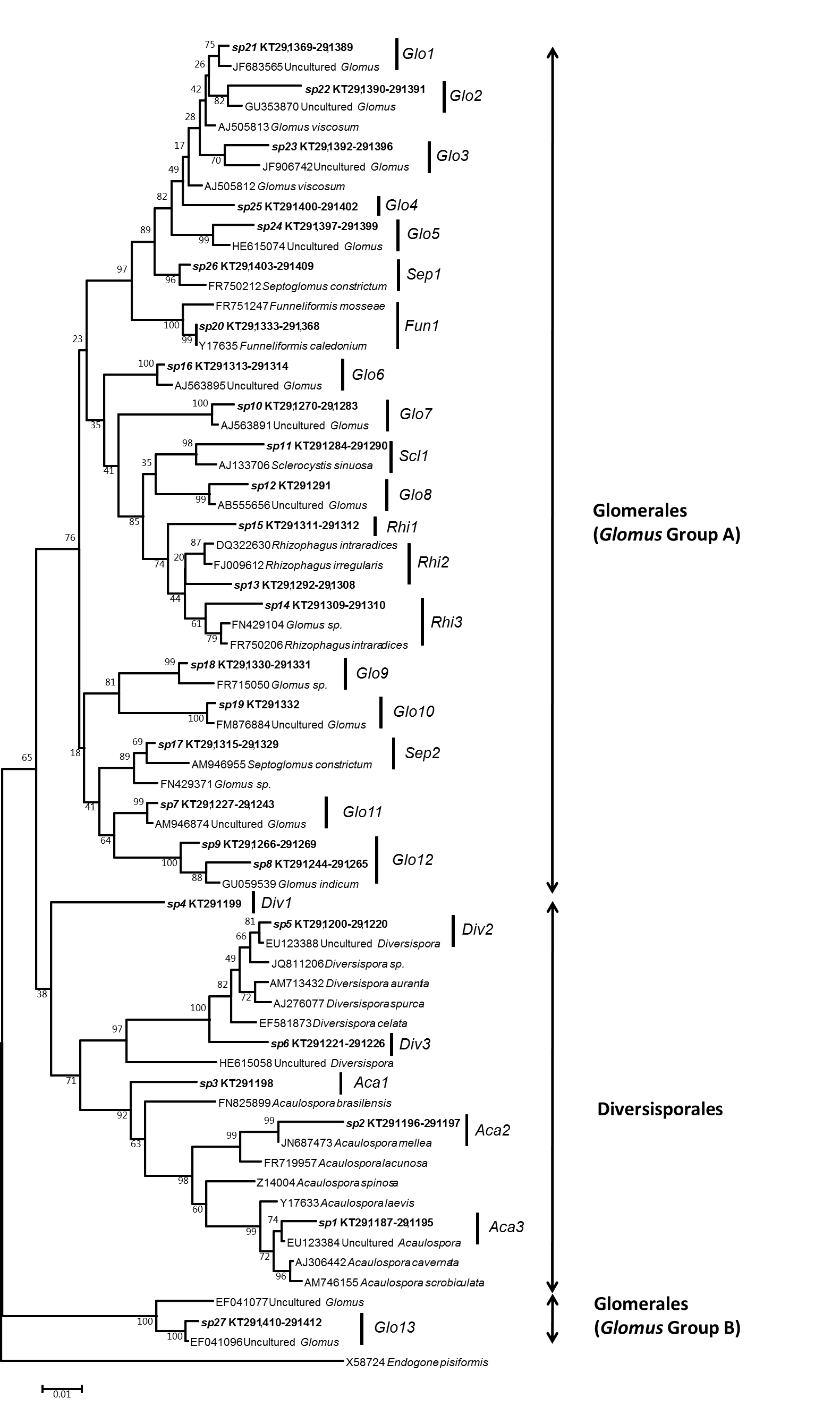


**Figure S3**

**Table** S1

| **Treatment** | **Soil depth**  **(cm)** | **Organic matter**  **(g kg^-1^)** | | **Total N**  **(g kg^-1^)** | **C/N** | **pH value** | **Available P**  **(mg kg^-1^)** | **N/P** | **Electrical conductivity**  **(EC) (μs cm^-1^)** | **Na**  **(mg kg^-1^)** | **Zn**  **(mg kg^-1^)** |
| --- | --- | --- | --- | --- | --- | --- | --- | --- | --- | --- | --- |
| **Control** | **0~20** | 12.32±0.28a | | 1.10±0.03a | 11.18±0.28ab | 8.35±0.06abc | 7.21±1.24c | 166.7 | 263.25±21.28d | 185.80±38.49d | 1.38±0.17a |
|  | **20~40** | 4.61±0.60b | | 0.72±0.01bc | 6.38±0.85d | 8.44±0.06a | 2.88±0.30c | 257.8 | 418.75±71.36cd | 518.80±154.09bcd | 0.85±0.13b |
|  | **40~60** | 4.90±0.48b | | 0.77±0.02bc | 6.35±0.66d | 8.21±0.09bcd | 2.61±0.31c | 307.8 | 828.50±172.25b | 859.80±108.89ab | 0.71±0.07b |
| **P25** | **0~20** | 13.52±0.46a | | 1.04±0.10a | 13.43±1.71a | 8.25±0.04abcd | 16.71±1.72b | 63.2 | 277.25±7.88d | 173.80±33.21d | 1.28±0.06a |
|  | **20~40** | 6.17±0.65b | | 0.83±0.09b | 7.50±0.29cd | 8.38±0.07ab | 3.83±0.16c | 219.4 | 286.00±29.40d | 290.05±67.28cd | 0.63±0.07b |
|  | **40~60** | 4.67±0.32b | | 0.65±0.03c | 7.21±0.24cd | 8.26±0.04abcd | 3.84±0.54c | 178.2 | 612.75±151.94bc | 686.05±241.24bc | 0.81±0.17b |
| **P100** | **0~20** | 13.03±1.08a | | 1.08±0.02a | 12.09±0.90a | 8.06±0.05d | 37.76±6.44a | 30.2 | 450.75±29.82cd | 246.80±56.81cd | 1.66±0.09a |
|  | **20~40** | 6.10±0.68b | | 0.70±0.03bc | 8.76±0.87bcd | 8.17±0.07cd | 7.16±3.15c | 142.8 | 708.75±111.26bc | 503.55±29.85bcd | 0.77±0.16b |
|  | **40~60** | 6.37±0.20b | | 0.69±0.02bc | 9.16±0.04bc | 8.16±0.05cd | 3.54±0.41c | 193.5 | 1311.00±213.20a | 1208.05±317.06a | 0.68±0.16b |
|  | | | **Analysis of variance** | | | | | | | | |
| **P fertilization** | | * | | ns | ns | ** | *** | ND | *** | ns | ns |
| **Soil depth** | | *** | | *** | *** | * | *** | ND | *** | *** | *** |
| **P fertilization × Soil depth** | | ns | | ns | ns | ns | *** | ND | ns | ns | ns |

Data are mean values±SE (n = 4). Significant differences among P treatments and soil depths within each variable were tested using Duncan's multiple range test (*p*<0.05) and are indicated by different letters. Two-way ANOVA was used to represent the main and interactive effects of P treatments and soil depths on each soil chemical property. Control: zero P application; P25: 25 kg P ha^-1^; P100: 100 kg P ha^-1^.* *p*<0.05; **

*p*<0.01; *** *p*<0.001; ns, non-significant. ND, not determined: N/P ratios are not normally distributed and it is therefore not valid to subject ratios to conventional parametric statistical tests.

**Table S**2

|  | **Phylotypes in roots** | | | | | | | | |  | **Phylotypes in soil** | | | | | | | | |
| --- | --- | --- | --- | --- | --- | --- | --- | --- | --- | --- | --- | --- | --- | --- | --- | --- | --- | --- | --- |
|  | **V6 stage** | | | **V13 stage** | | | **R4 stage** | | |  | **Control** | | | **P25** | | | **P100** | | |
|  | CK | P25 | P100 | CK | P25 | P100 | CK | P25 | P100 |  | 0-20cm | 20-40cm | 40-60cm | 0-20cm | 20-40cm | 40-60cm | 0-20cm | 20-40cm | 40-60cm |
| **97 bp** | **■** |  | **■** | **■** | **■** | **■** | **■** | **■** | **■** |  |  |  |  | **■** |  |  | **■** | **■** |  |
| **107 bp** | **■** |  |  |  |  |  | **■** |  |  |  |  |  |  | **■** |  |  |  |  |  |
| **116 bp** | **■** | **■** | **■** | **■** | **■** | **■** | **■** | **■** | **■** |  | **■** | **■** | **■** | **■** | **■** | **■** | **■** | **■** |  |
| **140 bp** |  |  |  |  |  |  |  |  |  |  |  |  |  | **■** |  |  | **■** | **■** |  |
| **141 bp** | **■** | **■** | **■** | **■** | **■** | **■** | **■** | **■** | **■** |  | **■** | **■** | **■** | **■** | **■** | **■** |  |  |  |
| **142 bp** |  |  |  |  |  |  |  |  |  |  |  |  |  | **■** |  |  |  |  |  |
| **157 bp** |  |  |  |  | **■** |  | **■** | **■** |  |  |  |  |  |  |  |  |  |  | **■** |
| **168 bp** |  |  |  | **■** |  |  | **■** | **■** |  |  |  |  |  | **■** |  |  | **■** |  |  |
| **169 bp** |  |  |  |  | **■** |  |  |  |  |  | **■** | **■** | **■** | **■** | **■** | **■** | **■** | **■** | **■** |
| **189 bp** | **■** | **■** |  | **■** | **■** | **■** | **■** | **■** | **■** |  | **■** |  | **■** | **■** |  |  | **■** | **■** | **■** |
| **190 bp** |  |  | **■** |  |  |  |  |  |  |  | **■** | **■** | **■** | **■** | **■** | **■** |  | **■** |  |
| **258 bp** | **■** | **■** |  | **■** | **■** | **■** | **■** | **■** | **■** |  |  |  |  | **■** | **■** |  | **■** | **■** |  |
| **259 bp** | **■** | **■** | **■** | **■** | **■** | **■** | **■** | **■** | **■** |  | **■** | **■** | **■** | **■** | **■** | **■** |  |  | **■** |

■ Presence of the T-RFs. CK: zero P application (control); P25: 25 kg P ha^-1^; P100: 100 kg P ha^-1^. V6 represents the six-leaf collar stage; V13 represents the thirteen-leaf collar stage; R4 represents the kernel dough stage.

**Table S3**

| **Phylogenetic lineage** | | | **97bp** | **107bp** | **116bp** | **140bp** | **141bp** | **142bp** | **157bp** | **168bp** | **169bp** | **189bp** | **190bp** | **191bp** | **258bp** | **259bp** | **Total** |
| --- | --- | --- | --- | --- | --- | --- | --- | --- | --- | --- | --- | --- | --- | --- | --- | --- | --- |
| **Diversisporales** | **Aca3** | sp1 |  |  |  |  |  | 9 |  |  |  |  |  |  |  |  | 9 |
|  | **Aca2** | sp2 | 2 |  |  |  |  |  |  |  |  |  |  |  |  |  | 2 |
|  | **Aca1** | sp3 |  |  |  |  |  | 1 |  |  |  |  |  |  |  |  | 1 |
|  | **Div1** | sp4 | 1 |  |  |  |  |  |  |  |  |  |  |  |  |  | 1 |
|  | **Div2** | sp5 |  |  |  |  | 3 |  |  |  | 17 |  |  |  |  | 1 | 21 |
|  | **Div3** | sp6 |  |  |  |  | 6 |  |  |  |  |  |  |  |  |  | 6 |
| **Glomerales** (*Glomus* Group A) | **Glo11** | sp7 |  |  |  | 17 |  |  |  |  |  |  |  |  |  |  | 17 |
|  | **Glo12** | sp8 |  |  |  |  |  |  |  |  |  | 22 |  |  |  |  | 22 |
|  | **Glo12** | sp9 |  |  |  |  |  |  | 2 |  |  | 2 |  |  |  |  | 4 |
|  | **Glo7** | sp10 |  |  |  |  |  |  |  | 13 |  |  | 1 |  |  |  | 14 |
|  | **Scl1** | sp11 |  | 7 |  |  |  |  |  |  |  |  |  |  |  |  | 7 |
|  | **Glo8** | sp12 |  |  |  |  |  |  |  |  |  |  |  |  | 1 |  | 1 |
|  | **Rhi2** | sp13 |  |  | 17 |  |  |  |  |  |  |  |  |  |  |  | 17 |
|  | **Rhi3** | sp14 |  |  |  |  | 2 |  |  |  |  |  |  |  |  |  | 2 |
|  | **Rhi1** | sp15 | 2 |  |  |  |  |  |  |  |  |  |  |  |  |  | 2 |
|  | **Glo6** | sp16 |  |  |  |  | 2 |  |  |  |  |  |  |  |  |  | 2 |
|  | **Sep2** | sp17 | 1 |  |  |  |  |  |  |  |  | 14 |  |  |  |  | 15 |
|  | **Glo9** | sp18 |  |  |  |  |  |  |  |  |  |  | 2 |  |  |  | 2 |
|  | **Glo10** | sp19 |  |  |  |  |  |  |  |  |  |  |  |  | 1 |  | 1 |
|  | **Fun1** | sp20 |  |  | 35 |  |  |  |  |  |  |  |  |  |  | 1 | 36 |
|  | **Glo1** | sp21 | 5 |  | 15 |  |  |  |  |  |  |  |  |  | 1 |  | 21 |
|  | **Glo2** | sp22 |  |  | 2 |  |  |  |  |  |  |  |  |  |  |  | 2 |
|  | **Glo3** | sp23 |  |  |  |  |  |  |  |  |  |  |  |  | 5 |  | 5 |
|  | **Glo5** | sp24 |  |  | 3 |  |  |  |  |  |  |  |  |  |  |  | 3 |
|  | **Glo4** | sp25 |  |  | 3 |  |  |  |  |  |  |  |  |  |  |  | 3 |
|  | **Sep1** | sp26 |  |  | 7 |  |  |  |  |  |  |  |  |  |  |  | 7 |
| **Glomerales** (*Glomus* Group B) | **Glo13** | sp27 |  |  |  |  |  |  |  |  |  |  |  | 3 |  |  | 3 |

**Table S4**

|  | Related species | Search against MaarjAM database | | | | |
| --- | --- | --- | --- | --- | --- | --- |
|  |  | Molecular virtual taxon | Agroecosystems | Forest | Grasslands | Total |
| **Diversisporales** | |  |  |  |  |  |
| sp1 Aca3 | *Acaulospora laevis* | VTX00030 | 0 | 1 | 2 | 3 |
| sp2 Aca2 | *Acaulospora mellea* | VTX00024 | 0 | 1 | 0 | 1 |
| sp3 Aca1 | *Acaulospora brasiliensis* | — | — | — | — | — |
| sp4 Div1 |  | — | — | — | — | — |
| sp5 Div2 |  | VTX00062 | 0 | 2 | 3 | 5 |
| sp6 Div3 | *Diversispora celata* | VTX00054 | 0 | 1 | 0 | 1 |
| **Glomerales (*Glomus* Group A)** | |  |  |  |  |  |
| sp7 Glo11 |  | VTX00130/00132/00175/00159/00140 | 2 | 5 | 1 | 8 |
| sp8 Glo12 | *Glomus indicum* | VTX00222/00125 | 4 | 2 | 1 | 7 |
| sp9 Glo12 | *Glomus indicum* | VTX00222/00125 | 2 | 2 | 2 | 6 |
| sp10 Glo7 |  | VTX00214 | 4 | 0 | 1 | 5 |
| sp11 Scl1 | *Sclerocystis sinuosa* | VTX00412/00069 | 2 | 2 | 1 | 5 |
| sp12 Glo8 |  | VTX00080/00361/00084 | 0 | 2 | 0 | 2 |
| sp13 Rhi2 | *Rhizophagus irregularis* | VTX00325/00295/00113/00100 | 0 | 3 | 2 | 5 |
| sp14 Rhi3 | *Rhizophagus intraradices* | VTX00100/00115 | 1 | 4 | 2 | 7 |
| sp15 Rhi1 |  | VTX00115 | 1 | 3 | 1 | 5 |
| sp16 Glo6 |  | VTX00202/00407/00103/00149/00148 | 1 | 3 | 0 | 4 |
| sp17 Sep2 | *Septoglomus constrictum* | VTX00156 | 5 | 2 | 1 | 8 |
| sp18 Glo9 |  | VTX00098 | 1 | 1 | 0 | 2 |
| sp19 Glo10 |  | VTX00419 | 2 | 3 | 0 | 5 |
| sp20 Fun1 | *Funneliformis caledonium* | VTX00065 | 5 | 1 | 4 | 10 |
| sp21 Glo1 | *Glomus viscosum* | VTX00063 | 2 | 4 | 0 | 6 |
| sp22 Glo2 | *Glomus viscosum* | VTX00063 | 2 | 2 | 0 | 4 |
| sp23 Glo3 | *Glomus viscosum* | VTX00063/00293 | 2 | 5 | 0 | 7 |
| sp24 Glo5 |  | VTX00409/00063 | 1 | 3 | 0 | 4 |
| sp25 Glo4 |  | VTX00063/00064/00409 | 2 | 3 | 0 | 5 |
| sp26 Sep1 | *Septoglomus constrictum* | VTX00064 | 0 | 4 | 1 | 5 |
| **Glomerales (*Glomus* Group B)** | |  |  |  |  |  |
| sp27 Glo13 |  | VTX00276/00225/00056 | 1 | 4 | 2 | 7 |
